# Supplementary material for: Nano-Based Approved Pharmaceuticals for Cancer Treatment: Present and Future Challenges
Source: Biomolecules. 2022 Jun 4;12(6):784. doi: 10.3390/biom12060784 (PMC9221343; doi:10.3390/biom12060784)
Supplement: Supplementary file 1 [file biomolecules-12-00784-s001.zip › biomolecules-1742917-supplementary.pdf]

Supplementary Table S1. Current active clinical trials using nanopharmaceuticals for cancer therapy in monotherapy or combined therapy.

NCT Number	Title	Indication	Intervention	Phase	Start Date	Refs.
NCT00662129	Paclitaxel Albumin-Stabilized Nanoparticle Formulation, Gemcitabine, and Bevacizumab in Treating Patients with Metastatic Breast Cancer	Breast Cancer	Biological: bevacizumab I Drug: gemcitabine hydrochloride I Drug: paclitaxel albumin-stabilized nanoparticle formulation	II	November 2008	162
NCT00110084	ABI-007 (Nab-Paclitaxel) and Gemcitabine in Treating Women with Metastatic Breast Cancer	Breast Cancer	Drug: Gemcitabine I Drug: Paclitaxel protein-bound particles for injectable suspension (albumin-bound)	II	August 2005	163
NCT00629499	Nanoparticle Albumin-Bound (Nab) Paclitaxel/Cyclophosphamide in Early-Stage Breast Cancer	Breast Cancer	Drug: nab paclitaxel I Drug: Cyclophosphamide I Drug: Trastuzumab	II	April 2008	164
NCT00856492	S0800, Nab-Paclitaxel, Doxorubicin, Cyclophosphamide, and Pegfilgrastim with or Without Bevacizumab in Treating Women with Inflammatory or Locally Advanced Breast Cancer	Breast Cancer	Biological: bevacizumab I Biological: pegfilgrastim I Drug: cyclophosphamide I Drug: doxorubicin hydrochloride I Drug: paclitaxel albumin-stabilized nanoparticle formulation	II	April 2010	165
NCT00553462	Carboplatin and Paclitaxel Albumin-Stabilized Nanoparticle Formulation Followed by Radiation Therapy and Erlotinib in Treating Patients with Stage III Non-Small Cell Lung Cancer That Cannot Be Removed by Surgery	Lung Cancer	Drug: carboplatin I Drug: erlotinib hydrochloride I Drug: paclitaxel albumin-stabilized nanoparticle formulation I Radiation: radiation therapy	II	March 2008	166
NCT00729612	Paclitaxel Albumin-Stabilized Nanoparticle Formulation and Carboplatin in Treating Patients with Stage IIIB, Stage IV, or Recurrent Non-Small Cell Lung Cancer	Lung Cancer	Drug: carboplatin I Drug: paclitaxel albumin-stabilized nanoparticle formulation I Genetic: protein expression analysis I Other: immunoenzyme technique I Other: immunohistochemistry staining method I Other: laboratory biomarker analysis	II	August 2008	167
NCT00675259	Phase II NCT (Neoadjuvant Chemotherapy) w/ Weekly Abraxane in Combination with Carboplatin & Bevacizumab in Breast Cancer	Breast Cancer	Drug: bevacizumab I Drug: carboplatin I Drug: nab-paclitaxel I Procedure: Surgery I Drug: Adjuvant chemotherapy	II	July 2008	168

NCT01620190	Paclitaxel Albumin-Stabilized Nanoparticle Formulation in Treating Patients with Previously Treated Advanced Non-small Cell Lung Cancer	Recurrent Non-Small Cell Lung Carcinoma, Stage IV Non-Small Cell Lung Cancer	Other: Laboratory Biomarker Analysis|Drug: Paclitaxel Albumin-Stabilized Nanoparticle Formulation	II	December 2012	169
NCT00691054	Abraxane Therapy in Patients with Pancreatic Cancer Who Failed First-Line Gemcitabine Therapy	Pancreatic Cancer	Drug: Abraxane	II	June 2008	170
NCT00616967	Carboplatin and Nab-Paclitaxel with or Without Vorinostat in Treating Women with Newly Diagnosed Operable Breast Cancer	Breast Cancer	Drug: carboplatin|Drug: paclitaxel albumin-stabilized nanoparticle formulation|Drug: vorinostat|Other: placebo	II	May 2008	171
NCT00407888	Doxorubicin Hydrochloride, Cyclophosphamide, and Filgrastim Followed by Paclitaxel Albumin-Stabilized Nanoparticle Formulation with or Without Trastuzumab in Treating Patients with Breast Cancer Previously Treated with Surgery	Several breast cancers	Drug: doxorubicin hydrochloride|Drug: cyclophosphamide|Biological: filgrastim|Drug: paclitaxel albumin-stabilized nanoparticle formulation|Biological: trastuzumab|Other: laboratory biomarker analysis|Procedure: quality-of-life assessment	II	May 2006	172
NCT00479674	Phase II Study with Abraxane, Bevacizumab and Carboplatin in Triple Negative Metastatic Breast Cancer	Breast Cancer	Drug: Abraxane|Drug: Bevacizumab|Drug: Carboplatin	II	May 2007	173
NCT02009332	Phase I/2 Study of ABI-009 in Nonmuscle Invasive Bladder Cancer	Non-muscle Invasive Bladder Cancer (NMIBC)	Drug: ABI-009|Drug: Gemcitabine	I/II	April 2014	174
NCT00733408	Nab-Paclitaxel and Bevacizumab Followed by Bevacizumab and Erlotinib in Metastatic Breast Cancer	Several breast cancers	Drug: paclitaxel albumin-stabilized nanoparticle formulation|Biological: bevacizumab|Drug: erlotinib hydrochloride	II	April 2008	175
NCT00466960	Sargramostim and Paclitaxel Albumin-Stabilized Nanoparticle Formulation in Treating Patients with Advanced Ovarian Cancer, Fallopian Tube Cancer, or Primary Peritoneal Cancer That Did Not Respond to Previous Chemotherapy	Brenner Tumor, Fallopian Tube Cancer, Peritoneal Cavity Cancer, many histological ovarian cancer	Biological: sargramostim|Drug: paclitaxel albumin-stabilized nanoparticle formulation|Other: laboratory biomarker analysis|Other: immunologic technique	II	May 2006	-
NCT00499252	Paclitaxel Albumin-Stabilized Nanoparticle Formulation in Treating Patients with Recurrent or	Fallopian Tube Carcinoma, Primary Peritoneal Carcinoma, Recurrent Ovarian Carcinoma	Drug: Paclitaxel Albumin-Stabilized Nanoparticle Formulation	II	June 2007	176

	Persistent Ovarian Epithelial Cancer, Fallopian Tube Cancer, or Primary Peritoneal Cancer					
NCT00785291	Paclitaxel, Nab-paclitaxel, or Ixabepilone with or Without Bevacizumab in Treating Patients with Stage IIIC or Stage IV Breast Cancer	Several breast cancers	Biological: Bevacizumab∣Drug: Ixabepilone∣Other: Laboratory Biomarker Analysis∣Drug: Nab-paclitaxel∣Drug: Paclitaxel∣Other: Questionnaire Administration	III	November 2008	177
NCT03670030	A Study to testABI-009 in Patients with Metastatic, Unresectable, Low or Intermediate Grade Neuroendocrine Tumors of the Lung or Gastroenteropancreatic System	Neuroendocrine Tumors	Drug: ABI-009	II	November 2018	-
NCT00093145	Study of Albumin-bound Paclitaxel (Abraxane) in Combination with Carboplatin and Herceptin in Patients with Advanced Breast Cancer	Breast Cancer	Drug: Albumin-bound paclitaxel∣Drug: Carboplatin∣Drug: HerceptinÂ®	II	June 2004	178
NCT00470548	Abraxane and Alimta in Advanced Solid Tumors	Breast Cancer, Lung Cancer, Unspecified Adult Solid Tumor, Protocol Specific	Drug: Abraxane∣Drug: Alimta	I/II	April 2007	179
NCT01938833	Romidepsin and Abraxane in Treating Patients with Metastatic Inflammatory Breast Cancer	Several breast cancers	Drug: Romidepsin∣Drug: Abraxane	I/II	April 2014	180
NCT00544648	Ph I/II Nab-Paclitaxel & Carboplatin w/Concurrent Radiation Therapy for Unresectable Stg III NSCLC	Lung Cancer	Drug: carboplatin∣Drug: nab-paclitaxel∣Radiation: Radiation therapy	I/II	November 2007	181
NCT00748553	A Phase I/II Clinical Trial of Vidaza with Abraxane in Patients with Advanced/Metastatic Solid Tumors and Breast Cancer	Advanced or Metastatic Solid Tumors, Advanced or Metastatic Breast Cancer	Drug: Azacitidine (Vidaza)∣Drug: Nab-paclitaxel (Abraxane)	I/II	September 2008	182
NCT01380769	A phase II Study of CRLX101(NLG207) in Patients with Advanced Non-Small Cell Lung Cancer	Non-Small Cell Lung Cancer	Drug: CRLX101∣Other: Best Supportive Care	II	June 2011	-
NCT00331630	Abraxane and Lapatinib in Treating Patients with Stage I, Stage II, or Stage III Breast Cancer	Breast Cancer	Drug: lapatinib ditosylate∣Drug: paclitaxel albumin-stabilized nanoparticle formulation	Early I	May 2006	183
NCT00851877	Nab-Paclitaxel, Cisplatin, and Cetuximab with Concurrent Radiation Therapy for Locally Advanced Head and Neck Cancer	Head and Neck Cancer	Biological: Cetuximab∣Drug: Cisplatin∣Drug: Nab-Paclitaxel∣Radiation: intensity-modulated radiation therapy	I/II	March 2009	184

NCT00709761	Phase II Lapatinib Plus Nab-Paclitaxel as First and Second Line Therapy In her2+ MBC	Neoplasms, Breast	Drug: Lapatinib/nab-Paclitaxel	II	July 2008	185
NCT01644890	A Phase III Study of NK105 in Patients with Breast Cancer	Breast Cancer Nos Metastatic Recurrent	Drug: NK105 I Drug: Paclitaxel	III	July 2012	186
NCT02562716	S1505: Combination Chemotherapy or Gemcitabine Hydrochloride and Paclitaxel Albumin-Stabilized Nanoparticle Formulation Before Surgery in Treating Patients with Pancreatic Cancer That Can Be Removed by Surgery	Pancreatic Adenocarcinoma, Resectable Pancreatic Carcinoma	Drug: Fluorouracil I Drug: Gemcitabine Hydrochloride I Drug: Irinotecan Hydrochloride I Drug: Oxaliplatin I Drug: Paclitaxel Albumin-Stabilized Nanoparticle Formulation I Procedure: Pancreatectomy	II	January 2016	187
NCT03120832	Phase I Trial of PAN-301-1 (SNS-301) in Cancer Patients	Prostate Cancer	Biological: PAN-301-1	I	December 2016	188
NCT01566435	Induction Chemotherapy with ACF Followed by Chemoradiation Therapy for Adv. Head & Neck Cancer	Head and Neck Neoplasms	Drug: paclitaxel albumin-stabilized nanoparticle formulation I Drug: Cisplatin I Drug: Fluorouracil I Radiation: Intensity modulated radiation therapy I Drug: Cetuximab I Procedure: Quality-of-life assessment	II	August 2012	189
NCT00821964	Topical Imiquimod and Abraxane in Treating Patients with Advanced Breast Cancer	Male Breast Cancer, Recurrent Breast Cancer, Skin Metastases, Stage IV Breast Cancer	Drug: imiquimod I Drug: Abraxane I Other: laboratory biomarker analysis I Genetic: RNA analysis I Other: immunoenzyme technique	II	December 2008	190
NCT01207102	Study Of Abraxane® And Carboplatin as First-Line Treatment for Triple Negative Metastatic Breast Cancer	Metastatic Breast Cancer	Drug: Abraxane I Drug: Carboplatin	II	August 2011	191
NCT01921751	High or Standard Intensity Radiation Therapy After Gemcitabine Hydrochloride and Nab-paclitaxel in Treating Patients with Pancreatic Cancer That Cannot Be Removed by Surgery	Pancreatic Adenocarcinoma, Stage III Pancreatic Cancer	Radiation: low intensity radiation therapy I Drug: Capecitabine I Drug: Gemcitabine I Radiation: high intensity radiation therapy I Drug: nab-Paclitaxel	II	August 2013	192
NCT03875092	A Study of Carboplatin-	Non-small Cell Lung Cancer	Biological: Pembrolizumab I Drug:	III	April	193

	Paclitaxel/Nab-Paclitaxel Chemotherapy with or Without Pembrolizumab (MK-3475) in Adults with First Line Metastatic Squamous Non-small Cell Lung Cancer (MK-3475-407/KEYNOTE-407)-China Extension Study		Paclitaxel∣Drug: Nab-paclitaxel∣Drug: Carboplatin∣Drug: Saline placebo for pembrolizumab		2017	
NCT02775435	A Study of Carboplatin-Paclitaxel/Nab-Paclitaxel Chemotherapy with or Without Pembrolizumab (MK-3475) in Adults with First Line Metastatic Squamous Non-small Cell Lung Cancer (MK-3475-407/KEYNOTE-407)	Non-small Cell Lung Cancer	Biological: Pembrolizumab∣Drug: Paclitaxel∣Drug: Nab-paclitaxel∣Drug: Carboplatin∣Drug: Saline placebo for pembrolizumab	III	June 2016	194
NCT02608229	BVD-523 Plus Nab-paclitaxel and Gemcitabine in Patients with Metastatic Pancreatic Cancer	Pancreatic Cancer, Cancer of Pancreas, Cancer of the Pancreas, Pancreas Cancer	Drug: BVD-523∣Drug: Nab-paclitaxel∣Drug: Gemcitabine∣Procedure: Tumor biopsy	I	June 2016	195
NCT02258659	Nab-paclitaxel and Carboplatin Followed by Response-Based Local Therapy in Treating Patients with Stage III or IV HPV-Related Oropharyngeal Cancer	Human Papilloma Virus Infection, Stage III, IVA and IVB Squamous Cell Carcinoma of the Oropharynx	Drug: paclitaxel albumin-stabilized nanoparticle formulation∣Drug: carboplatin∣Radiation: radiation therapy∣Drug: paclitaxel∣Drug: fluorouracil∣Drug: hydroxyurea∣Drug: cisplatin∣Other: laboratory biomarker analysis∣Procedure: quality-of-life assessment	II	September 2014	196
NCT01746225	Schedules of Nab-Paclitaxel in Metastatic Breast Cancer	Metastatic Breastcancer	Drug: nab-Paclitaxel	II	April 2013	197
NCT00309959	ABI-007 in Treating Patients with Persistent or Recurrent Cervical Cancer	Many histological cervical cancers	Drug: Paclitaxel Albumin-Stabilized Nanoparticle Formulation∣Other: Laboratory Biomarker Analysis	II	November 2006	198
NCT03101358	Study of Topical SOR007 Ointment for Cutaneous Metastases	Cutaneous Metastasis	Drug: SOR007 (Uncoated Nanoparticle Paclitaxel) Ointment	I/II	January 2018	199
NCT01646762	Paclitaxel Albumin-Stabilized Nanoparticle Formulation in Treating Patients with Relapsed or Refractory Multiple Myeloma	Refractory Plasma Cell Myeloma	Drug: Paclitaxel Albumin-Stabilized Nanoparticle Formulation∣Other: Laboratory Biomarker Analysis	II	November 2012	200
NCT02010567	Neoadjuvant Chemoradiotherapy With CRLX-101 and Capecitabine for Rectal Cancer	Rectal Cancer	Drug: CRLX101∣Drug: Capecitabine∣Radiation: Radiotherapy∣Procedure: Surgery	I/II	December 2013	201

NCT00254592	Neoadjuvant Treatment of Breast Cancer	Breast Cancer	Drug: Doxorubicin丨Drug: Cyclophosphamide丨Drug: Carboplatin丨Drug: Nab-paclitaxel丨Drug: GM-CSF丨Drug: Trastuzumab丨Drug: Bevacizumab	II	October 2005	202
NCT00626405	Bevacizumab and Temozolomide or Bevacizumab and Paclitaxel Albumin-Stabilized Nanoparticle Formulation and Carboplatin in Treating Patients with Stage IV Malignant Melanoma That Cannot Be Removed by Surgery	Melanoma (Skin)	Biological: bevacizumab丨Drug: carboplatin丨Drug: paclitaxel albumin-stabilized nanoparticle formulation丨Drug: temozolomide	II	August 2008	203
NCT02427841	Nab-paclitaxel and Gemcitabine Hydrochloride Followed by Radiation Therapy Before Surgery in Treating Patients with Pancreatic Cancer That Can Be Removed by Surgery	Pancreatic Adenocarcinoma, Resectable Pancreatic Carcinoma	Drug: Fluorouracil丨Drug: Gemcitabine丨Radiation: Image Guided Radiation Therapy丨Radiation: Intensity-Modulated Radiation Therapy丨Other: Laboratory Biomarker Analysis丨Drug: Nab-paclitaxel丨Procedure: Therapeutic Conventional Surgery	II	January 2016	-
NCT00738361	Paclitaxel Albumin-Stabilized Nanoparticle Formulation in Treating Patients with Metastatic Melanoma of the Eye That Cannot Be Removed by Surgery	Intraocular Melanoma	Drug: nab-paclitaxel	II	August 2008	204
NCT00404235	Carboplatin and ABI-007 in Treating Patients with Stage IV Melanoma That Cannot Be Removed by Surgery	Melanoma (Skin)	Drug: carboplatin丨Drug: paclitaxel albumin-stabilized nanoparticle formulation	II	October 2006	205
NCT02314052	Phase Ib/2, Multicenter, Dose Escalation Study of DCR-MYC in Patients with Hepatocellular Carcinoma	Hepatocellular Carcinoma	Drug: DCR-MYC	I/II	January 2015	-
NCT02626520	Phase II Evaluation of Multi-modality Algorithm for Non-metastatic Adenocarcinoma of Pancreas or Ampulla	Ductal Adenocarcinoma of Pancreas, Adenocarcinoma of Ampulla	Drug: Gemcitabine and nanoparticle albumin bound paclitaxel丨Drug: 5-fluorouracil and irinotecan丨Radiation: Preoperative chemoradiation丨Procedure: Definitive resection	II	May 2016	206

NCT02340156	Phase II Study of Combined Temozolomide and SGT-53 for Treatment of Recurrent Glioblastoma	RECURRENT GLIOBLASTOMA	Genetic: SGT-53 I Drug: Temozolomide	II	December 2014	-
NCT02158520	Nab-Paclitaxel and Bevacizumab or Ipilimumab as First-Line Therapy in Treating Patients with Stage IV Melanoma That Cannot Be Removed by Surgery	Melanomas	Biological: Bevacizumab I Biological: Ipilimumab I Other: Laboratory Biomarker Analysis I Drug: Nab-paclitaxel I Other: Pharmacological Study	II	October 2013	207
NCT04240639	An Extension Study MRI/US Fusion Imaging and Biopsy in Combination with Nanoparticle Directed Focal Therapy for Ablation of Prostate Tissue	Neoplasms of the Prostate	AuroShell particle infusion	Not Applicable	January 2020	208
NCT04138342	Topical Fluorescent Nanoparticles Conjugated Somatostatin Analog for Suppression and Bioimaging Breast Cancer	Breast Cancer<br>Skin Cancer	Drug: Quantum dots coated with veldoreotide	I	December 2022	209
NCT04789486	Nano-SMART: Nanoparticles With MR Guided SBRT in Centrally Located Lung Tumors and Pancreatic Cancer	Lung Cancer<br>Pancreatic Cancer	Drug: AGuIX<br>Injected gadolinium-based nanoparticles<br>Radiation: Radiotherapy<br>Stereotactic magnetic resonance-guided adaptive radiation therapy	I	May 2021	-
NCT05010759	Study of Focal Ablation of the Prostate With NanoTherm® Therapy System for Intermediate-Risk Prostate Cancer	Prostate Cancer	Device: NanoTherm Ablation	Not Applicable	November 2021	-
NCT05359783	Sentinel Node Localization and Staging With Low Dose Superparamagnetic Iron Oxide (MAGSNOW)	Breast Cancer<br>Sentinel Lymph Node	Drug: Superparamagnetic Iron Oxide	I/II	November 2021	210
NCT04316091	A Phase I Clinical Trial of Neoadjuvant Chemotherapy With/Without Superparamagnetic Iron Oxide Nanoparticles and Spinning Magnetic Field for Patients With Osteosarcoma	Osteosarcoma	Drug: neoadjuvant chemotherapy+SPIONs/SMF<br>Intratumoral injection of SPIONs, followed by SMF, combined with conventional neoadjuvant chemotherapy	I	August 2021	-
